# Study on the prediction of prognosis of patients with acute liver failure treated with artificial liver by serum NLR and IL-6 levels

**DOI:** 10.5937/jomb0-57618

**Published:** 2025-10-28

**Authors:** Juan Xu, Fenjing Du, Xin Yang, Jingtao Hou, Yan Fan, Xiaojing Liu

**Affiliations:** 1 The First Affiliated Hospital of Xi'an Jiao Tong University, Department Infectious Disease, Xian, Shaanxi, 710061, China

**Keywords:** acute liver failure, artificial liver support system, neutrophil/lymphocyte ratio, interleukin-6, predictive performance, akutna insuficijencija jetre, sistem podrške veštačke jetre, neutrofilno-limfocitni odnos, interleukin-6, prediktivna vrednost

## Abstract

**Background:**

To observe the relationship between the expression of serum interleukin-6 (IL-6) and neutrophil/lymphocyte ratio (NLR) and the prognosis of patients with acute liver failure (ALF) treated with artificial liver.

**Methods:**

80 patients with ALF from January 2021 to October 2023 were included. All of them received artificial liver system treatment. All of the included subjects completed the effective follow-up for three months. The patients were followed up until April 30, 2023. According to the disease outcomes of patients after the end of follow-up, they were divided into a survival group and a death group. The serum IL-6 and NLR expressions of the two groups were compared, and the relationship between serum IL-6 and NLR expressions and the prognosis of patients with ALF treated with artificial liver was analysed.

**Results:**

The expression levels of serum IL-6 and NLR in the death group were higher (P&lt;0.05). The high expressions of serum IL-6 and NLR might be a risk factor for the increased risk of death in patients with ALF after treatment (P&lt;0.05). The receiver operating characteristic (ROC) curve indicated that the area under the curve (AUC) of fasting serum IL-6 and NLR expressions in patients with ALF predicting the prognosis of artificial liver treatment were 0.727 and 0.789. When the AUC of serum IL-6 combined with NLR predicted the prognosis of artificial liver treatment in patients with ALF, it was 0.889.

**Conclusions:**

The expression of serum IL-6 and NLR is correlated with the prognosis of patients with ALF treated with artificial liver.

## Abstract (sr)

**Uvod:**

Posmatranje odnosa između ekspresije interleukina-6 (IL-6) u serumu i neutrofilno-limfocitnog odnosa (NLR) sa prognozom pacijenata sa akutnom insuficijencijom jetre (ALF) sa veštačkom jetrom.

**Metode:**

U istraživanje je uključeno 80 pacijenata sa ALF u periodu od januara 2021. do oktobra 2023. Svi su primili terapiju putem sistema veštačke jetre. Svi ispitanici su uspešno praćeni tokom tri meseca, a praćenje je trajalo do 30. aprila 2023. Na osnovu ishoda bolesti nakon završetka praćenja, pacijenti su podeljeni u grupu preživelih i grupu preminulih. Upoređene su vrednosti IL-6 i NLR u serumu između ove dve grupe, te je analiziran njihov odnos sa prognozom pacijenata sa ALF lečenih veštačkom jetrom.

**Rezultati:**

Vrednosti IL-6 i NLR u serumu su bile značajno više u grupi preminulih pacijenata (P&lt;0,05). Povišeni nivoi IL-6 i NLR mogu da predstavljaju faktore rizika za povećanu smrtnost kod pacijenata sa ALF nakon lečenja (P&lt;0,05). ROC kriva je pokazala da je površina ispod krive (AUC) za predviđanje prognoze lečenja veštačkom jetrom na osnovu nivoa IL-6 i NLR iznosila 0,727 i 0,789. Kada su kombinovani nivoi IL-6 i NLR, AUC je iznosila 0,889.

**Zaključak:**

Ekspresija IL-6 i NLR u serumu povezana je sa prognozom pacijenata sa ALF lečenih veštačkom jetrom.

## Introduction

Acute liver failure (ALF) is a critical disease with high morbidity and mortality [1]. An artificial liver support system (ALSS) is an external detoxification device that can temporarily assist and replace part of the function of the failing liver [2]. It has the function of removing harmful substances such as urea and bilirubin in the body, helping to stabilise the environment and promoting liver cell regeneration [3]. As a bridge waiting for liver transplantation and liver function recovery, it is currently an effective and profitable treatment for ALF [4]. However, approximately 30-40% of ALF patients still progress to multiorgan failure post-ALSS treatment, underscoring limitations in addressing systemic inflammation and predicting long-term outcomes [5]. Consequently, refining prognostic tools to guide therapeutic decisions remains an urgent unmet need.

Recently, prognostic evaluation in ALF increasingly recognises the pivotal role of systemic inflammation in disease progression. Inflammatory cascades drive circulatory collapse, tissue hypoperfusion, and secondary organ damage, with interleukin-6 (IL-6) – a key proinflammatory cytokine– directly correlating with hepatic necrosis severity and mortality [6]. Concurrently, the neutrophil-to-lymphocyte ratio (NLR), a readily measurable hematologic marker, reflects systemic inflammatory burden and immune dysregulation, showing prognostic value in sepsis and liver diseases [7] [8]. Notably, IL-6 induces neutrophilia and suppresses lymphocyte proliferation, mechanistically linking it to NLR elevation [9]. While both markers independently predict outcomes in ALF, their combined utility in assessing ALSS efficacy remains unexplored. Existing studies focus predominantly on single-marker analysis or post-transplant outcomes [10] [11], leaving a critical gap in understanding how dynamic inflammatory interplay influences ALSS-specific recovery.

This study innovatively investigates the synergistic relationship between serum IL-6 and NLR in ALF patients undergoing ALSS therapy. By elucidating their combined prognostic capacity, we aim to establish a novel dual-biomarker model that enhances early risk stratification. This approach holds significant clinical promise as it may enable personalised adjust ments to ALSS protocols, timely escalation to transplantation, and, ultimately, improved survival rates in this high-risk population.

## Materials and methods

### Included subjects

First, we estimated the sample size using the PASS software. Eighty patients with ALF from January 2021 to October 2023 were included. The study was conducted with the consent of the Medical Ethics Committee (No. E2021024). All enrolled subjects met the following inclusion conditions: (1) Inclusion criteria: ①The diagnosis of ALF met the requirements of the Guideline for diagnosis and treatment of liver failure [12]: no-precipitating jaundice deepened on the basis of chronic liver disease, and the serum total bilirubin exceeded ten times of normal level/the daily increase value 17.1 μmol/L; Coagulation disorders: prothrombin activity <40%/international standard ratio 1.5, bleeding tendency, and combination with extrahepatic failure symptoms such as hepatic encephalopathy [13]; ②The participants were psychologically and spiritually normal and could cooperate with the research; ③The guardian of the enrolled subject knows the research purpose and signs the consent form. (2) Exclusion criteria: ①Accompanied with liver cancer, alcohol disease, autoimmune disease, drug-induced hepatitis and other diseases; ②Accompanied with HIV, virus and other liver diseases; ③Accompanied with metabolic diseases; ④Accompanied with decreased functions of heart, kidney and other organs; ⑤Patients were treated with hormones and liver protection before entering the group.

### Methods

### Data collection

A general data questionnaire is used to collect general demographic information about patients and relevant clinical examinations, including basic data such as gender, age and hepatic encephalopathy.

### Laboratory examination

5 mL of fasting peripheral blood was collected from included subjects after admission to the hospital. The blood sample was placed into an EDTA-K2 anticoagulation tube. The following indicators were detected using an XE-2100 blood cell analyser and supporting facilities provided by SYSMEX Corporation in Japan: absolute neutrophil count (ANC), and the normal value was (1.8–6.3)×10^9^/L; absolute lymphocyte count (ALC), the normal value was 20.0%–40.0%; NLR=ANC/ALC. The blood samples were centrifuged at 3000 r/min for 15 minutes. Serum IL-6 level was detected using ELISA, and the operation was carried out strictly according to the instructions of the kit (purchased from Wuhan Baiyixin Biotechnology Co., Ltd., Lot No.: TD711391).

### Follow-up and grouping

All the enrolled subjects completed the effective follow-up for three months. Follow-up visits were conducted by hospital review at least once a month. The follow-up date ended on April 30, 2024.

### Statistical method

SPSS24.0 was used to process the data. The Kolmogorov-Smirnov test was used to test whether the measurement data conformed to the normal distribution, and »x̄±s« was used to indicate the measurement data conformed to the normal distribution. The t-test was used to compare the independent samples between groups. The count data were expressed as percentages using the χ^2^ test. Logistic regression analysis was used to test the correlation between serum IL-6 and NLR expressions and the prognosis of patients with ALF after treatment (Hosmer-Lemeshow). The Receiver Operating Characteristic (ROC) of subjects was drawn to test the value of serum IL-6 and NLR expressions in predicting the prognosis of patients with ALF after artificial liver treatment, evaluated by the area under curve (AUC), predictive value details were shown in Table 1, with the test level α=0.05.

**Table 1:** AUC predictive value details.

AUC	Predictive value
≤0.50	No
0.50<AUC≤0.70	Low
0.70<AUC≤0.90	Medium
>0.90	High

## Results

### Prognosis

At the end of the follow-up of 80 patients with ALF, 50 patients survived, with a survival rate of 62.50% in the survival group; 30 patients died of illness, with a mortality rate of 37.50% in the death group.

### Comparison of general data

Comparing the general data between the two groups (*P*>0.05). The expression levels of serum IL-6 and NLR in the death group were higher ( *P* <0.05) (Table 2).

**Table 2:** Comparison of general data.

Factor		Death group<br>(n=30)	Survival group<br>(n=50)	Statistical values	P
Gender n (%)	Male	18(60.00)	32(64.00)	*χ^2^* =0.128	0.721
	Female	12(40.00)	18(36.00)		
Age (x̄±s, years)		45.10±5.25	45.84±5.52	*t*=0.591	0.556
Child-Pugh classification<br>n (%) [14]	Grade A	10(33.33)	8(16.00)	*χ^2^* =3.231	0.072
	Grade B	20(66.67)	42(84.00)		
Hepatic encephalopathy<br>n (%) [15]	Yes	12(40.00)	28(56.00)	*χ^2^* =1.920	0.166
	No	18(60.00)	22(44.00)		
Serum IL-6 (x̄±s, pg/mL)		31.95±9.35	23.85±6.80	*t*=4.471	<0.001
NLR (x̄±s)		7.25±2.25	3.68±1.02	*t*=9.708	<0.001

### Logistic regression analysis

The serum IL-6 and NLR expressions of patients with ALF were regarded as covariates, and the prognosis of the artificial liver in patients with ALF was regarded as a dependent variable (0=survival group, 1=death group). The Logistic regression model was established, and the equation was ob tained: y=-9.007+0.106 (IL-6)+0.995 (NLR), the high expres sions of serum IL-6 and NLR might be a risk factor for the increased risk of death in patients with ALF after treatment (*P*<0.05) (Table 3).

**Table 3:** Logistic regression analysis.

Variable	* B *	* S.E *	* Wals *	* P *	*OR*<br>value	95%<br>confidence interval	
						Upper limit	Lower limit
Constant	-9.007	2.054	19.239	<0.001	0.000	-	-
IL-6	0.106	0.043	5.907	0.015	1.112	1.021	1.210
NLR	0.995	0.223	19.893	<0.001	2.459	1.747	4.190

### Efficacy analysis of serum IL-6 and NLR expressions in predicting the prognosis of patients with ALF treated with artificial liver

**Figure 1:**
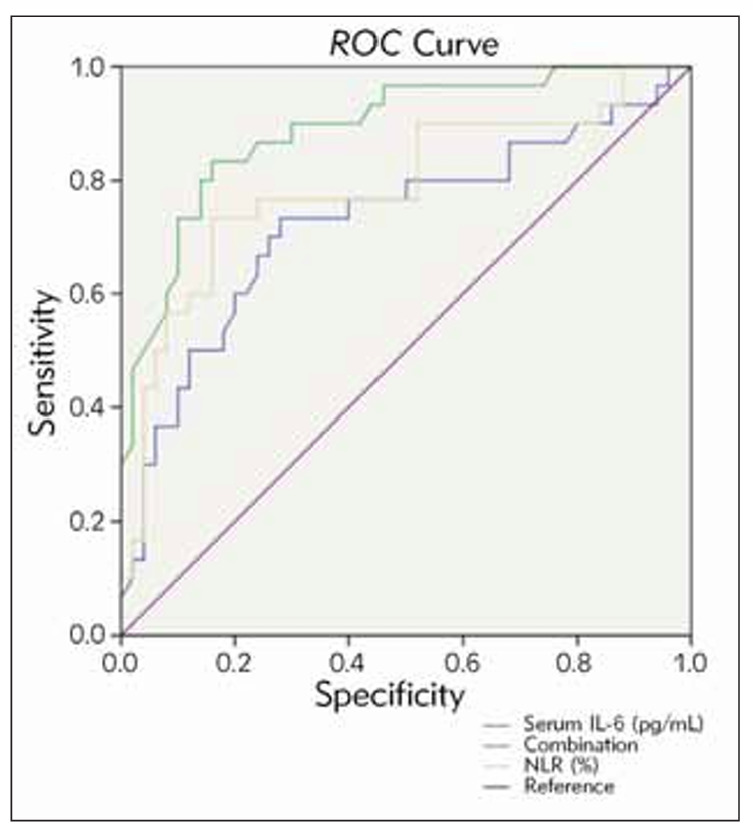
ROC curve of serum IL-6 and NLR expressions predicting the prognosis of patients with ALF treated with artificial liver.

The serum IL-6 and NLR expressions in patients with ALF were taken as test variables, and the prognosis of patients with ALF treated with artificial liver was taken as a state variable (0=survival group, 1=death group). ROC curve was drawn (Figure 1). The ROC curves showed that the AUC of fasting serum IL-6 and NLR expression in ALF patients for predicting prognostic death from artificial liver therapy was 0.727 and 0.789, respectively. The AUC of serum IL-6 in combination with NLR for predicting prognostic death from artificial liver therapy in ALF patients was 0.889, which was a highly favourable predictive value (Table 4).

**Table 4:** Efficacy analysis of serum IL-6 and NLR expressions in predicting the prognosis of patients with ALF treated with artificial liver.

Indicator	AUC	95% CI<br>of AUC	Standard<br>error	* P *	Cut-off<br>value	Specificity	Sensitivity	Youden index
IL-6	0.727	0.605–0.850	0.063	0.001	11.905	0.960	0.967	0.927
NLR	0.789	0.678–0.900	0.057	0.000	3.310	0.740	0.967	0.707
Combination	0.889	0.815–0.964	0.038	0.000	-	0.720	0.967	0.687

The AUC of serum IL-6 in combination with NLR for predicting prognostic death from artificial liver therapy in ALF patients was 0.889.

## Discussion

In this study, we found that serum IL-6 and NLR were strongly associated with the prognostic condition of ALF patients after ALSS, and these findings provide new references for future clinical optimisation of clinical treatment of ALF.

The systemic inflammatory response has been confirmed to be associated with the prognosis of patients with ALF [14] [15] [16] [17]. The release of cytokines, infection, immune dysfunction, and oxidative stress can drive the systemic inflammatory response. IL-6, a pro-cytokine with the presence of T lymphocytes, macrophages, and monocytes, can activate the host defence function in a short time to maintain liver tissue homeostasis and regeneration capacity [18] [19]. The serum IL-6 expression in the death group was higher in this study, which might be due to the following reasons: after liver failure, the liver function was damaged, and the body developed a cascade of inflammatory reactions, in which IL-6 was rapidly synthesised and released into the blood, and the lymphocytes were necrotic due to the gradually aggravating inflammatory reaction, which caused the significant increase of inflammatory factors, and the consequent increase in IL-6 expression [20] [21] [22]. In a study by Ye Q et al. on pan-cellular inflammatory death in ALF, they similarly confirmed that IL-6 is elevated in ALF [23], consistent with our results. NLR is the ratio of neutrophils to lymphocytes, and these two main cellular components belong to natural and adaptive immunity. The former mainly mediates the inflammatory response pathway, while the latter participates in the immune regulatory pathway. Immunoregulation and inflammatory response are important factors that cannot be ignored in the occurrence of ALF and disease progression [24] [25]. Wang N et al. [26] also mentioned in their study that NLR has the potential to be a short-term prognostic assessment index for chronic liver failure, which confirms our view. The results of this study indicated that the level of NLR in patients of the death group was higher, and the reason may be: ① After ALF, a large number of hepatocytes necrosis and neutrophil activation participated in the inflammatory reaction. On the one hand, activated neutrophils released a large amount of protease, which led to the aggravation of hepatocyte injury while clearing the infected cells. On the other hand, active oxygen produced by neutrophils led to cell lipid peroxidation and indirectly injured hepatocytes. These necrotic hepatocytes secrete a large number of cellular products, such as interleukin-17, which act as chemokines to promote the release of neutrophils in the hepatic sinusoids into the blood, causing increased expression of neutrophils in serum [27] [28]. ② The decreased expression of lymphocytes is also one of the factors that cause high expression of NLR. When ALF occurs, the inflammatory response of the body is excessively activated, and the expression of inflammatory factors in serum is increased, resulting in massive consumption and necrosis of lymphocytes. Secondly, lymphocytes, especially activated and differentiated T cells, are recruited into the liver and participate in the inflammatory response of the liver, leading to the decreased expression of lymphocytes in peripheral blood so that the NLR in peripheral blood is significantly increased.

To verify the above assumption, the Logistic regression model further obtained the equation in this study: y=-9.007+0.106 (IL-6)+0.995 (NLR). The high expressions of serum IL-6 and NLR might be a risk factor for the increased risk of death in patients with ALF after treatment. The results of ROC indicated that the AUC of fasting serum IL-6 and NLR expressions in patients with ALF for predicting the prognosis of artificial liver treatment were 0.727 and 0.789. When the AUC of serum IL-6 combined with NLR for predicting the prognosis of artificial liver treatment in patients with ALF was 0.889, the predictive value was ideal. These results suggested that the levels of serum IL-6 and NLR could be used as biological indicators for predicting the prognosis of artificial liver treatment in patients with ALF. The possible reasons for analysis were as follows: ① After ALF, damage-associated molecular patterns (DAMP) caused the activation of a large number of liver macrophages and released inflammatory factors. In the early stage of inflammation, various factors led to a large number of hepatocyte necrosis, triggered the local inflammatory organs, and induced a waterfall inflammatory reaction. At the same time, liver macrophages released a large number of inflammatory factors, such as tumour necrosis factor and IL-6 [29] [30]. ② A large amount of inflammatory cells and their release of proinflammatory factors, including IL-6, will lead to abnormal changes such as hepatocyte necrosis and liver fibrosis. Liver stellate cells will be activated to damage hepatocytes, resulting in abnormal changes in liver microcirculation, increased portal pressure, impaired liver self-repair and regeneration function. After persistent hepatocyte injury, a large number of inflammatory factors will be further released, leading to systemic inflammatory response syndrome, immune paralysis, and others, and eventually organ failure and increased risk of death for patients [31]. ③ The expression of neutrophils increases, which accelerates the activation of inflammation and simultaneously releases a large number of granular enzymes. For example, myeloperoxidase can directly damage oxidised tissues; Elastase participates in the maturation induction of IL-1 and induces the inflammatory cascade reaction, which directly damages the histiocytes and mediates the occurrence of insulin resistance. In addition, the decline of phagocytosis of immune cells and portosystemic shunt results in the increase of endotoxin cycle, aggravating the circulatory disturbance of liver failure, as well as the decline of organ and tissue perfusion, thus increasing the risk of death [32].

However, because of the small included sample size and short follow-up period, the conclusion of the study is biased, and in the future, we need to expand the sample size, extend the follow-up period and other means to further study the mechanism of serum IL-6 and NLR in the treatment and prognosis of patients with ALF.

## Conclusion

The expression of serum IL-6 and NLR is correlated with the prognosis of patients with ALF treated with artificial liver. The high expression of serum IL-6 and NLR may be a risk factor for the increased risk of death in patients with ALF after treatment. In the future, clinical attention should be focused on patients with high expression of serum IL-6 and NLR, thus reducing the risk of patient death after artificial liver therapy.

## Dodatak

### Availability of data and materials

The data used to support the findings of this study are available from the corresponding author upon request.

### Funding

No funds, grants, or other support were received.

### Acknowledgements

Not applicable.

### Authors’ contributions

XJ. L. conceived and designed the study. J. X. and FJ. D. wrote and revised the manuscript, X. Y. and JT. H. collected and analysed data, Y. F. supervised the study, and J. X. and FJ. D. made equal contributions to this work as co-first authors. All authors read and approved the final submitted manuscript.

### Conflict of interest statement

All the authors declare that they have no conflict of interest in this work.
